# Wood anatomy and dendrochronological potentiality of some woody shrubs from the southern Mediterranean coast in Egypt

**DOI:** 10.3389/fpls.2023.1183918

**Published:** 2023-06-28

**Authors:** Emad A. Farahat, Holger Gärtner

**Affiliations:** ^1^Botany and Microbiology Department, Faculty of Science, Helwan University, Cairo, Egypt; ^2^Forest Dynamics, Dendrosciences, Swiss Federal Research Institute (WSL), Birmensdorf, Switzerland

**Keywords:** subtropical shrubs, Mediterranean, growth rings, X-ray density, wood anatomy, dendroecology

## Abstract

In tropical and subtropical regions, much research is still required to explore the dendrochronological potential of their trees. This study aims to evaluate the anatomical structure and dendrochronological potential of three Mediterranean desert shrubs in Egypt (*Lycium schweinfurthii* var. *schweinfurthii*, *L. europaeum*, and *Calligonum polygonoides* subsp*. comosum*) supported by X-ray density. The results showed that the target species had distinct growth rings at macroscopic and microscopic levels. The vessel traits reflected the adaptability of each species with the prevailing arid climate conditions. After the exclusion of the non-correlated series, we obtained three site chronologies that cover the years 2013-2022 for *L. schweinfurthii*, 2012-2022 for *L. europaeum*, and 2011-2022 for *C. comosum*. The mean series intercorrelation was 0.746, 0.564, and 0.683 for *L. schweinfurthii*, *L. europaeum*, and *C. comosum*, respectively. The EPS (expressed population signal) values ranged from 0.72 to 0.80, while the SNR (species-to-noise ratio) ranged from 9.1 to 21.5. Compiling all series of *L. schweinfurthii* raised the EPS value to 0.86. The chronologies developed for the studied species were relatively short since we dealt with multi-stemmed shrubs. The average percentage difference between latewood density (LWD) and earlywood density (EWD) in *C. comosum*, *L. europaeum*, and *L. schweinfurthii* were 11.8% ± 5.5, 5.2%± 1.87, and 3.6% ± 1.86, respectively. X-ray densitometry helped in the precise determination of the ring borders of the studied species. The relationships between the radial growth of the studied species and the climate variables were weak to moderate but mostly not significant (i.e., r < 0.7). Generally, the radial growth of the target species had a weak to moderate positive correlation with temperature and precipitation during the wet season (winter), while negatively correlated with temperature for the rest of the year, particularly in summer. Our data agrees with earlier findings that ring formation starts at the beginning of the long vegetative stage, then the rest of the assimilated carbohydrates are directed to the flowering and fruiting at the end of the vegetative stages. For more efficient dendrochronological studies on subtropical and Mediterranean trees, we recommend carrying out xylogenesis studies, collection of phenological data, sampling 45-80 trees per species, using new techniques, and choosing homogeneous and close sites for wood sampling.

## 1 Introduction

Tropical and subtropical lands are the most biologically diverse, productive, and poorly studied environments in the world (Corlett, 2018; Deharveng and Bedos, 2019). However, dendrochronologists have long shied away from them due to the frequently inconsistent growth patterns of tropical trees, challenging sampling conditions, complex wood anatomy, and/or a lack of physiological understanding of local wood species (Pearl et al., 2020; Rodriguez et al., 2022). Recently, for tropical and sub-tropical tree species, studying tree growth by using the annual tree rings is becoming essential for more understanding of the ecological performance of trees (Farahat and Gärtner, 2019; Quesada-Román et al., 2022).

In tropical, subtropical, and Mediterranean climates, not all plants can develop detectable xylem rings. This is due to the less pronounced seasonality in climate conditions, which renders ring borders distinctness to be unreliable. Additionally, changes in the growth season’s climate result in false rings, wedging rings, and intra-annual density variations in wood (Rozendaal and Zuidema, 2011; Balzano et al., 2019). The results of many investigations (e.g., Haines et al., 2018; Farahat et al., 2022; García-López et al., 2022) support the difficulty of dendrochronological studies conducted in subtropical regions, where the complexity of ring boundary distinctness and tree-ring analysis is greatly exacerbated by a combination of genetic and environmental factors, such as the diversity of habitats and local variations in climate seasonality. A significant knowledge gap on the response of North-African plants to climate change at the individual tree and population level still exists (Zuidema et al., 2013; Zhao et al., 2019). The annual growth rings can be used to gauge how trees are reacting to climate change. Consequently, many ecological concerns about the nature of tree growth can be addressed if the environmental and growth data are properly extracted from tree rings (Farahat et al., 2022; Rodriguez et al., 2022).

Up to now, numerous species have been proven to be suitable for dendrochronological study in tropical and subtropical regions. However, there is still work to be done to separate the climatic and biological information present in tropical and subtropical plants (Quesada-Román et al., 2022). There are currently many technologies and approaches used to help in improving our understanding of the response of these plants to environmental conditions. Among these approaches are wood anatomy, isotopes, X-ray densitometry, and X-ray fluorescence either separately or in combination (e.g., Haines et al., 2018; Mihaljevič et al., 2018; Gaitan-Alvarez et al., 2019; Bai et al., 2021; Pacheco-Solana et al., 2023). These technologies proved their usefulness in dendrochronological studies and improved our understanding of the climate-growth relationship in trees and their ecophysiology.

Though, this study aims to evaluate the dendrochronological potential of three Mediterranean desert multi-stemmed, deciduous shrubs in Egypt (*Lycium schweinfurthii* var. *schweinfurthii* Dammar, *L. europaeum* L., and *Calligonum polygonoides* subsp*. comosum* L’ Hér.) using multi-proxy techniques (wood anatomy and X-ray densitometry). This will include macroscopic and microscopic examination of their wood and identification of their growth ring characteristics. We hypothesize that since the three species experience a withering period during the dry and hot summer, the formation of annual rings will be influenced by the prevailing climate conditions and their phenology under the southern Mediterranean conditions.

## 2 Materials and methods

### 2.1 Study species

This study includes three perennial desert shrubby species: *Lycium schweinfurthii* var. *schweinfurthii* Dammar, *Lycium europaeum* L. (Solanaceae), and *Calligonum polygonoides* subsp*. comosum* L’ Hér. (Polygonaceae). With roughly 97 species, the *Lycium* genus is a significant part of the Solanaceae family. *L. schweinfurthii* is a member of the Solanaceae family that is naturally distributed along the African coastal area, Sicily, Cyprus, and Crete (Boulos, 2002). Its leaves and fruits are used in traditional medicine due to its contents of alkaloids, glycosides, sterols, saponins, resins, phenolics, and flavonoids (Mamdouh and Smetanska, 2022). Although the limited distribution areas, Shaltout et al. (2018) reported that this species is distributed in many habitats (coastal sand dunes, flat sand sheets, roadsides, island sand dunes, and canal banks) along the Mediterranean coast in Egypt.

*Lycium europaeum* is a widely distributed deciduous shrub in the dry, sandy soils of the Mediterranean Basin. In Egypt, it is a profusely branched shrub, widely distributed in the Mediterranean and Sinai, and occupies wider areas compared to the previous species (Bedair et al., 2020). The species is known for its medicinal importance (Tej et al., 2018). The species is suggested as a potential candidate for stabilizing movable dunes and slopes that have deteriorated due to harsh environmental conditions (Touati et al., 2017). The two *Lycium* species are woody, spiny, multi-stemmed perennial shrubs that can reach 4 meters in height. *L. europaeum* that we collected at our sampling sites had a bigger crown area, with many robust woody stems in contrast to *L. schweinfurthii*.

*Calligonum polygonoides* subsp*. comosum* (*C. comosum*, hereafter) is commonly distributed in North Africa, Southern Europe, and Western Asia. The plant inhabits sandy soil and dune habitats (Boulos, 1999). This species may reach 2 m in height and lacks the main trunk. It is characterized by many wood branches. The plant is widely used in traditional medicine and its extracts showed many medical applications (Ahmed et al, 2020; Alzahrani, 2021). In addition, *C. comosum* has a positive influence on the stability of the desert ecosystem, at least by stabilizing sand through its far-reaching and rather dense root system (Al-Farraj, 1989; Taia and Moussa, 2011).

### 2.2 Site characteristics

**Figure 1:**
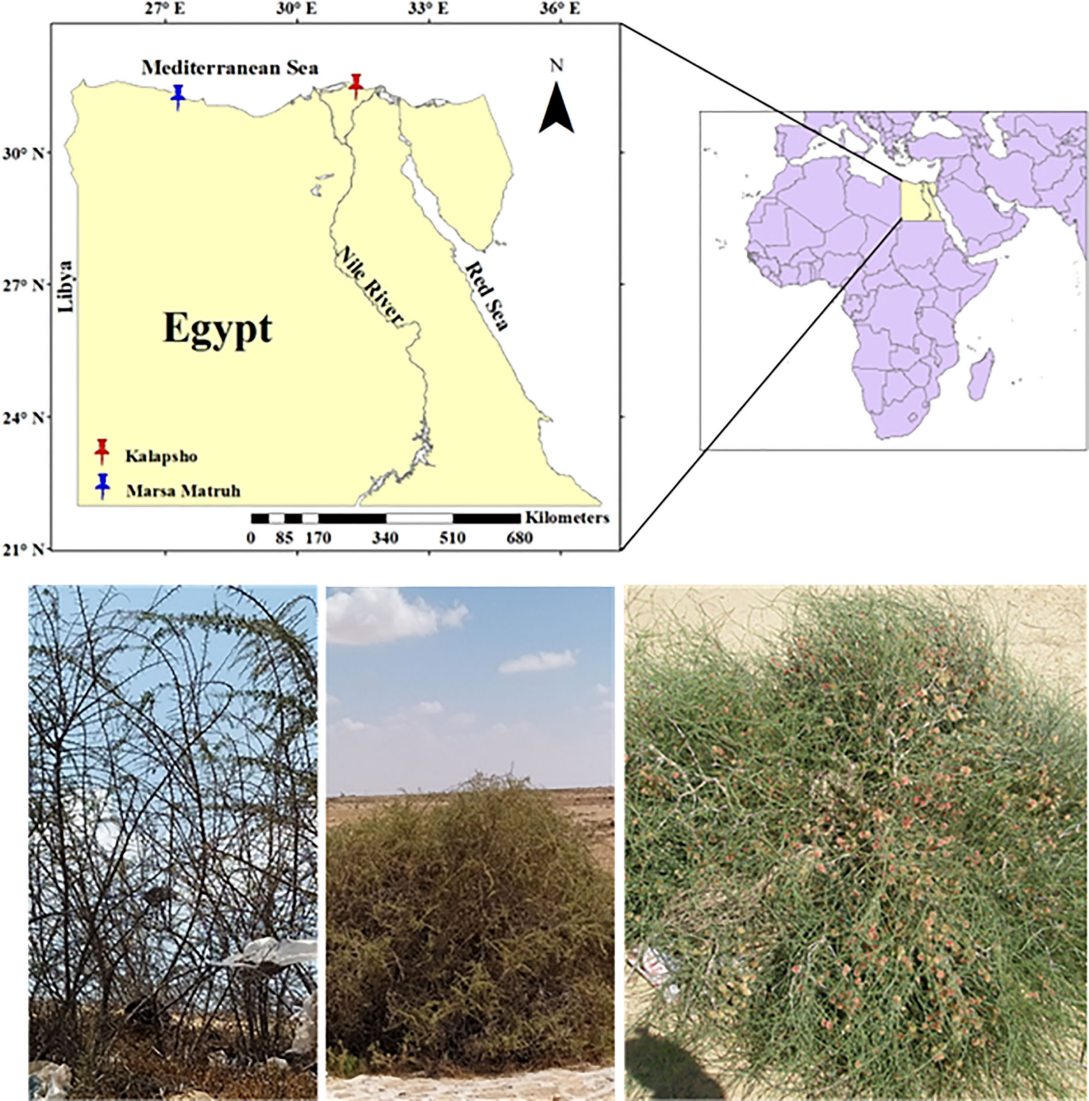
Map showing the studied locations for the target species, from left to right: *Lycium schweinfurthii*, *Lycium europaeum*, and *Calligonium comosum*.

**Figure 2:**
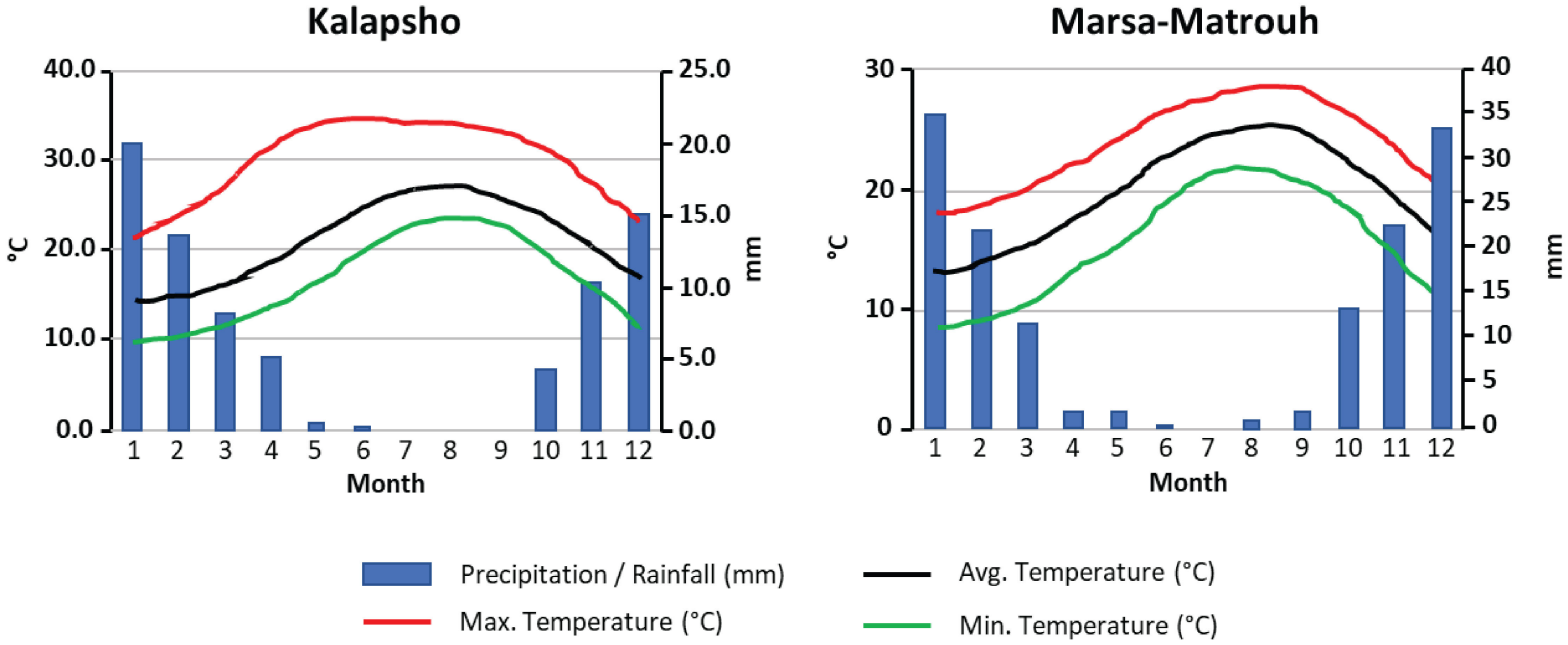
Climate diagrams for the studied locations show the monthly mean, maximum, and minimum temperatures (°C), and mean monthly precipitation (mm).

All samples were collected in late June 2022 during the withering stage (Beshara, 2019). *L. schweinfurthii* and *C. comosum* were collected at Kalapsho, Dakahlia Governorate (31°28’54.63” N, 31°19’29.68” E) on dunes and sandy soil habitats, about 3-6 km from the coast (**Figure 1**). The samples of *L. europaeum* were collected at Marsa Matruh, New Valley Governorate, Egypt (31°14’35.45”N, 27°17’27.99”E) on sandy and gravel sandy soil, about 1.5-9 km from the coast (**Figure 1**). At Kalapsho location, the warmest month is August with an average temperature of 27.7 °C, reaching a maximum of 34.8 °C, while the coldest month was February with an average temperature of 14.9 °C (**Figure 2**). The total annual precipitation was 78.0 mm/year with an average of 6.5 mm/month. The summer months (June-August) are rainless. At the Marsa Matruh location, August was the warmest month with an average (maximum) temperature of 25.3 °C (28.5 °C). The coldest month was January with an average temperature (minimum) of 13.1 °C (8.4 °C) (**Figure 2**). The total annual precipitation was 150 mm/year with an average of 12.5 mm/month. It is worth noting that there is no precipitation in the summer months (June-August). January is the wettest month on both sites while the summer months are, despite being the driest, the warmest. Climate data for the studied sites were downloaded from NASA Prediction Of Worldwide Energy Resources (NASA POWER, https://power.larc.nasa.gov/).

### 2.3 Sample collection, processing, and wood anatomy

Wood samples were collected for the target species by cutting stem discs (3-4 cm thickness) from the shrubs on site. At each location, we sampled wood discs from many sites. The final numbers of stem discs used for further analysis after excluding some of them due to bad structure or the presence of dark stains in wood were 18 and 15 discs for *L. schweinfurthii* and *L. europaeum*, respectively. Since the presence of aged wood shrubs of *C. comosum* is not so common in the Egyptian deserts, we found and successfully collected 9 aged stem discs from nine different shrubs, but we used eight for further analysis after excluding one disc. The wood discs were sanded to get a smooth surface and visible rings for macroscopic examination. Micro-sections (thickness 15–20 μm) were prepared from the same wood for microscopic analysis and ring-width measurements using the WSL-lab-microtome (Gärtner and Farahat, 2021). For each sample, at least three replicas were made, and the micro-sections were stained with safranin and astra-blue, dehydrated with ethanol, embedded in Canada Balsam, and then oven-dried in an oven at 60°C for 24 h (Gärtner and Schweingruber, 2013). A Zeiss slide scanner Axio Z1 was used to capture high-resolution micro-photos at 100x magnification. The digital images were then used to visually determine the characteristics of the ring boundaries for all species. The image analysis program WinCELL (ver. 2020a, Regent Instruments, Canada) was utilized to measure the vessel traits (total vessel lumen area%, mean total vessel lumen area, vessel density, and average cell lumen) in each tree ring from the micro-photos. During measurement, a suitable filter was applied in the program’s settings to omit all additional cells, including fibers.

### 2.4 X-ray density measurements

Along the same radii we defined to cut the microsections, X-ray densitometry was measured. The wood samples were cut carefully using a twin-bladed saw to obtain ca. 1.5mm thick laths along the radii. Following Kaczka et al. (2018), a climate-controlled environment (20°C, 50% relative humidity) was used to create an analog X-ray image of each sample. Using a Balteaugraph X-ray generator, the samples were exposed to X-rays for 70 minutes at 12 kV/17 mA, and the image was recorded on fine-grained film paper (Kodak Industrex). Finally, based on the analog X-ray images, a Walesch Dendro 2003 densitometry workstation was utilized to create and measure wood density (Eschbach et al., 1995). A cellulose acetate multi-stepped calibration wedge with known material density and step depths was simultaneously X-rayed. In the final phase, a calibration factor of 0.886 that was empirically determined was used to scale the grey level to gravimetric densities (Schweingruber, 1988). The Walesch system’s step resolution is provided by a measuring sensor width of 0.5 mm. The real measurement consequently produces 20 and 10 µm step resolution because the grey level measurement profiles are collected at 25- and 50-time magnification, respectively. The following information was obtained from tree-ring density profiles for each annual ring: earlywood (EW) and latewood widths (LW), minimum (MND), and maximum wood densities (MXD hereafter). Because earlywood and latewood mean densities and MND and MXD are closely connected, we only examined the EW and LW because they are simpler to define and exhibit a stronger response to climate variables (Camarero et al., 2014). Following Camarero et al. (2017), we utilized a 50% level between the MND and MXD values of each ring to establish the earlywood-latewood transition.

Most recently, Björklund et al. (2021) pointed out that microdensitometric techniques such as X-ray densitometry are used as a standard in tree-ring research, but their accuracy has been questioned. Consequently, there is a need to verify the accuracy of microdensitometric measurements and even correct them using additional techniques such as bulk wood density measurements, which are defined as gravimetric/volumetric wood density (Williamson and Wiemann, 2010). To measure bulk wood density, the same wood samples used for preparing laths for X-ray densitometry were weighed to the nearest centigram and the bulk volume was determined by water displacement (Hill and Papadopoulos, 2001) following the protocol presented by Björklund et al. (2021) by dividing the sample weight by the sample volume.

### 2.5 Ring-width measurement

Using the scanned microsections of wood samples, the tree-ring widths were measured to the nearest 0.001mm using Cybis™ CooRecorder and CDendro software (Cybis Electronics, Sweden). Two radii for each sampled tree were measured when if there was no eccentric growth or wedging rings. TSAP-Win (Rinn, 2010) and COFECHA (Holmes, 1983) software were used to perform and verify both visual and statistical cross-dating. A reference chronology for each tree was developed after omitting all poorly correlated time series. Using average mean sensitivity and average inter-series correlation, cross-dating quality was evaluated. Mean sensitivity is defined as “the measure of the relative difference in the width of adjacent rings and is used as an indicator of climate sensitivity in the tree ring record” (Fritts, 2001). The average inter-series correlation is “the average of all Pearson’s correlation coefficients calculated for each tree-ring series against the composite chronology once the series being tested is removed” (Holmes, 1983; Grissino-Mayer, 2001). The tree-ring data were detrended to enhance the typical growth variability related to climatic conditions while removing the growth trend (Fritts, 2001). Due to the data being short and because we were interested in retaining the growth dynamics of the individual trees, linear detrending of the reference chronologies was calculated before correlating it with linear detrended climate data. Linear detrending was done using R (R Core Team, 2013). For the standardized TRW chronologies, signal-to-noise ratio (SNR) and expressed population signal (EPS) were calculated (Wigley et al., 1984). The EPS measures how closely a given sample chronology resembles the idealized perfect chronology, which may therefore be viewed as a potential climate signal; an acceptable statistical value has been empirically suggested as a threshold of 0.85. SNR is an expression of the strength of the observed common signal among trees. For *L. schweinfurthii*, it was not easy to deal with its tree series in Cdendro or TSAP because some of them were too short. Accordingly, and to increase the sample depth of the reference chronology, after building the first reference chronology from considerably older tree series, we carried out a Pearson correlation analysis between the young time series and the reference chronology. Then, we compiled all young series with correlation coefficient (r) > 0.65 to the reference chronology and we build an additional chronology for all highly correlated series.

### 2.6 Data analysis

The relationships between the standardized TRW chronologies of the target species and climate data (monthly average temperature T and total monthly precipitation P) for the current growing season were analyzed. The correlation with the climatic data of the previous season was excluded due to the high autocorrelation values in TRW and climatic data. Pearson’s correlation coefficients between the detrended TRW and the current growing season climate were calculated. The same analysis was done between the detrended TRW and vessel traits. One-way ANOVA was carried out to compare the annual variations in vessel traits for the studied species using SPSS software (ver. 21).

## 3 Results

### 3.1 Wood anatomical characteristics

**Figure 3:**
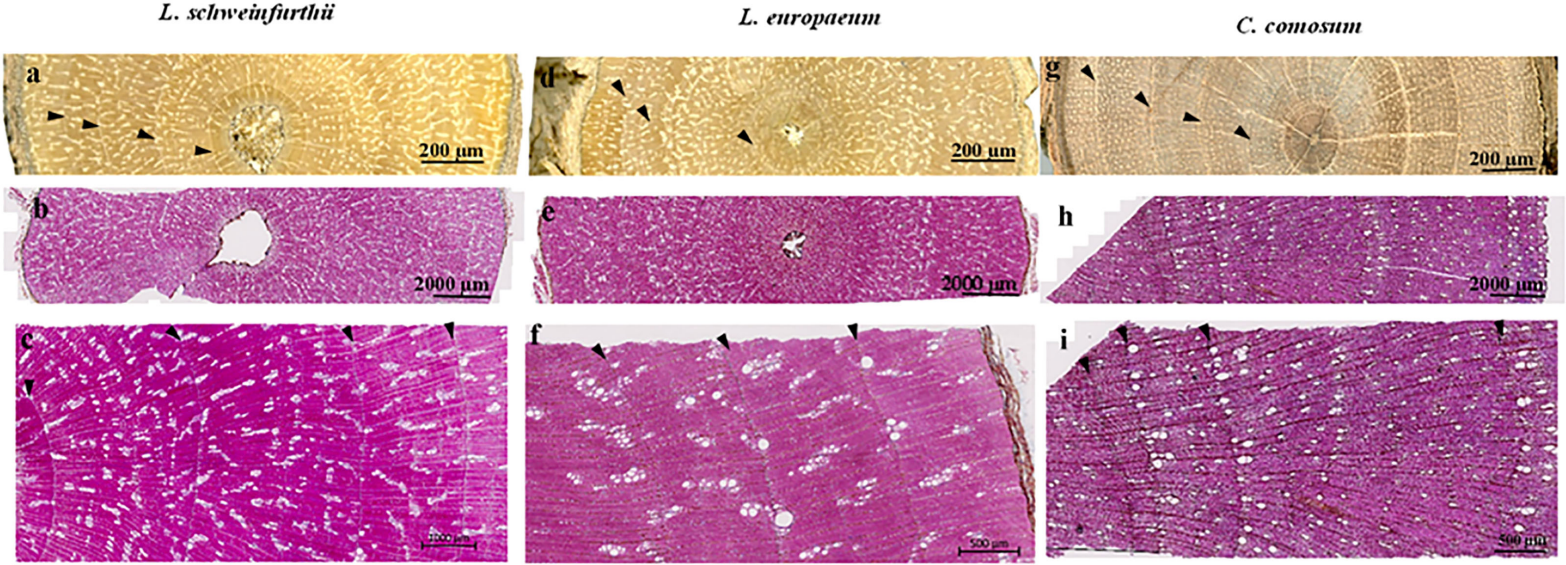
Acroscopic wood anatomy of the studied species **(A, D, G)** and the anatomical transverse sections showing the ring boundaries **(B, C, E, F, H, I).** The arrows refer to ring boundaries.

**Figure 4:**
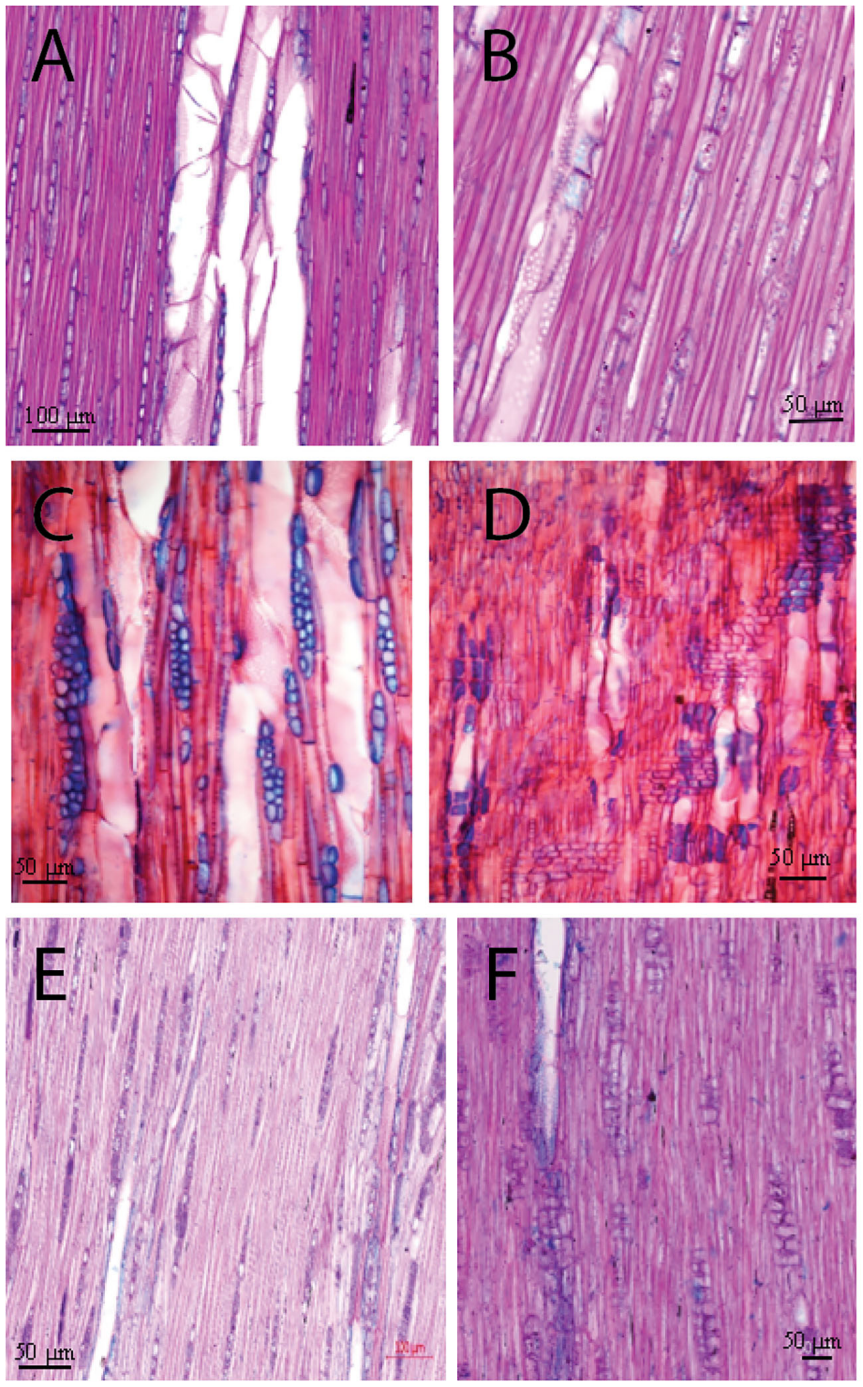
Tangential and radial micro-section of the target species: *L. schweinfurthii* **(A, B)**, *L. europaeum* **(C, D)**, and *C. comosum* **(E, F)**. AP, axial parenchyma; R, rays; V, vessel.

Macroscopically, the wood of *L. europaeum* is semi-ring porous (**Figures 3D–F**). The wood is pale yellowish brown with bright yellowish-white dendritic or radial structures that represent areas predominantly occupied by vessels. At a microscopic level, ring boundaries are distinct and determined by a very thin layer of flattened fibers with brown secretions or large parenchyma-like cells (mostly one cell in thickness) embedded in them. Furthermore, the semi-ring porous character of the rings supports the determination of the ring boundaries by the change in vessel size in the earlywood of the next ring. The onset of the ring is characterized by groups of relatively big vessels with an average diameter of 45µm, frequently arranged in radial files. Vessels are round to oval; in the latewood portion of the ring they are arranged diagonally to radial or in a dendritic pattern and embedded in thick-walled fibers. Vessels commonly occur in 1-4 radial rows as short multiples. Axial parenchyma is scanty apotracheal or paratracheal. The rays show dark brown secretions like infills or dark solitary crystals inside. Tangentially, rays are uni- and biseriate, with a nucleus in each cell. The vessels do show simple perforation plates. Intervessel pittings are simple, oval to round, sometimes bordered, alternate and opposite. Vessel-ray pits are large and round. Ray cells are upright and squared (**Figures 4A, B**).

Macroscopically, there is no significant difference in the color of *L. schweinfurthii* wood and *L. europaeum*. A minor difference is that the bright areas occupied by vessels are smaller in size compared to *L. europaeum* (**Figures 3A–C**). Microscopically, wood is semi-ring porous. Ring boundaries are indicated by the gradual change in vessel diameters and a single row of thick-walled fibers with dark secretions/crystals and small parenchyma-like cells embedded in them. Vessels are arranged in a diagonal to radial or in a dendritic pattern, embedded in thick-walled fibers. The vessel pattern could vary from one ring to the other in the same sample. Vessels commonly occur in 1-4 rows as short or long multiples. Earlywood vessels show an average diameter of 77.8 µm. Axial parenchyma is scanty apotracheal or paratracheal. The rays are uniseriate with a nucleus in some cells. Perforation plates are simple. Intervessel pits are simple, oval to round, alternate, and opposite. Vessel-ray pits are large and round. Ray cells are upright and squared (**Figures 4C, D**).

The wood of *C. comosum* is ring-porous with distinct rings. Macroscopically, the wood is faint to dark brown with bright pale brown areas occupied by vessels that accumulate adjacent to the ring borders, visually indicating yellowish-white ring boundaries (**Figures 3G–I**). Microscopically, ring boundaries are characterized by a tangential layer of thin-walled parenchyma cells (3-7 rows) embedded in thick-walled fibers and the presence of the earlywood vessels of the next ring. Earlywood vessels are usually confined to the first part of the ring adjacent to the ring border while latewood vessels occupy a large area of the ring. Vessels are mostly solitary or in short multiples (2-4), round to oval. The average diameter of the earlywood vessels is 80.0 µm. Fibers are thick-walled. Axial parenchyma is commonly paratracheal vasicentric to aliform, present commonly as marginal bands above the ring boundaries. Rays are uniseriate and lignified. Dark-staining substances are present in the pith. Rays are uni- to biseriate, lignified and short containing dark-staining substances. Perforation plates are simple. Intervessel pits are simple, round in alternating positions, and polygonal. Vessel-ray pits are simple with distinct borders. Ray cells are upright and squared. Slit-like or round pits are present in the radial walls of fibers (**Figures 4E, F**).

### 3.2 Wood density characteristics

**Figure 5:**
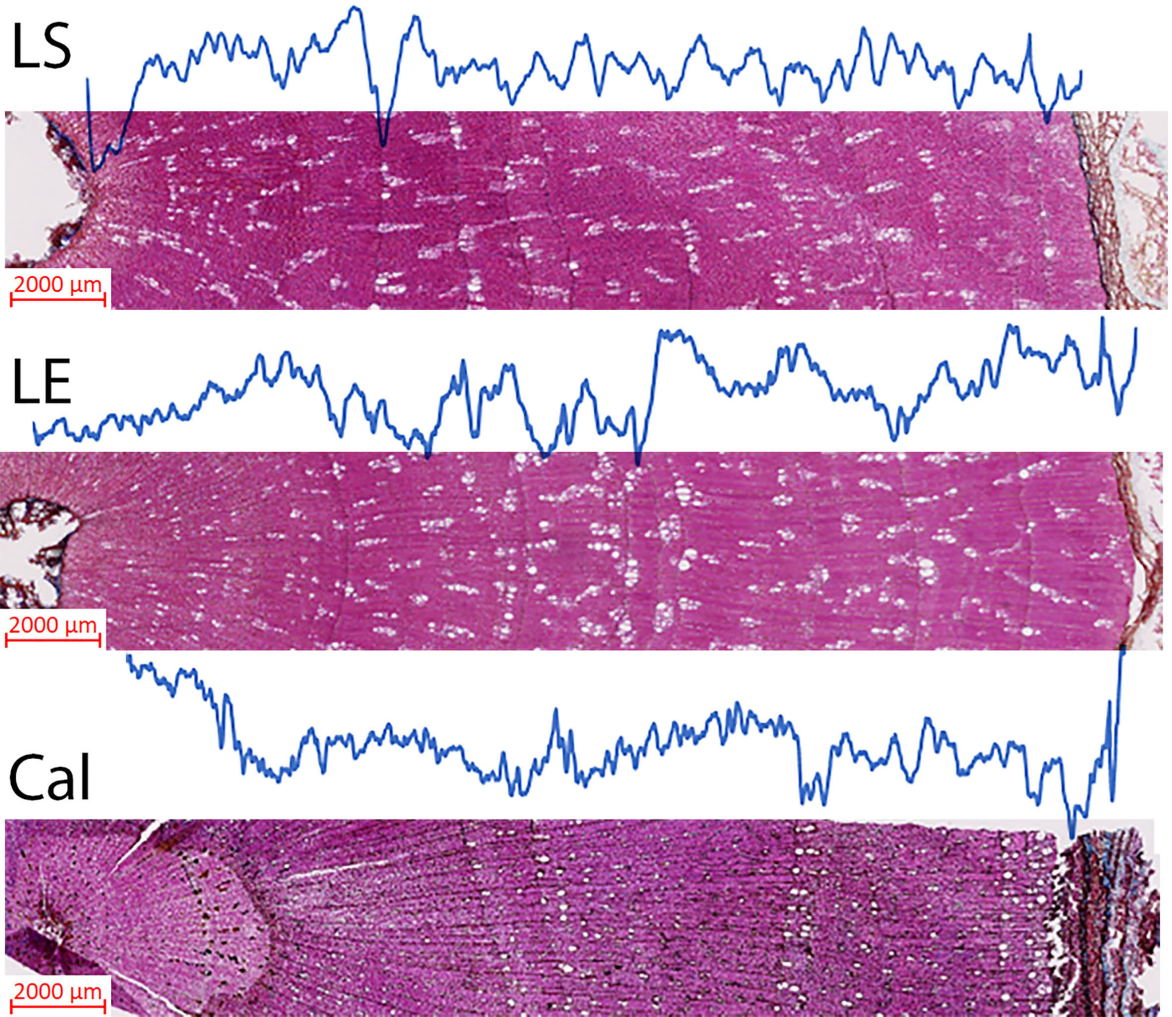
Complete X-ray wood density profiles for three samples representing the studied species. Measurements were taken under x25 magnification. The real measurement consequently produces a 20 µm step resolution. LS, *L. schweinfurthii*; LE, *L. europaeum*; and Cal, *C. comosum*.

Bulk wood density and X-ray density measurements for the studied species were carried out (**Figure 5**; **Table 1**). The mean bulk wood density ( ± SD) was 664 (± 37.30), 709 (± 38.54), and 704 (± 30.82) (g/dm^3^) for *L. europaeum, L. schweinfurthii*, and *C. comosum*, respectively. On the other hand, the mean corrected X-ray density values for the same species were 698.09 ± 4.06, 751.12 ± 59.86, and 847.5± 40.45 g/dm^3^, respectively. The relationships between bulk wood density and the X-ray density were relatively strong with R^2^ equal to 0.62, 0.75, and 0.82 for *L. europaeum, L. schweinfurthii*, and *C. comosum*, respectively. The average percentage difference between latewood density (LWD) and earlywood density (EWD) in *C. comosum* was 11.8% ± 5.5. On the other hand, the LWD and EWD in *L. europaeum* were 735.55 ± 12,96 g/dm^3^ and 704.81 ± 21.9 g/dm^3^, respectively. In *L. schweinfurthii* LWD and EWD were 780.17 ± 32.48 g/dm^3^ and 750.20± 29.60 g/dm^3^, respectively. In *L. europaeum* and *L. schweinfurthii*, the average percentage difference between LWD and EWD was 5.2%± 1.87 and 3.6% ± 1.86, respectively. The decrease in wood density values was progressive and observed at the ring boundary (i.e., end of the latewood). These decreases sometimes become more abrupt when there are many earlywood vessels next to the ring boundary.

**Table 1:** Wood density of the target species and the relationship between bulk wood density and X-ray density.

Species	no. of samples	Bulk wood density (g/dm^3^)	Mean X-ray Density (g/dm^3^)	Mean Calibrated X-ray Density (g/dm^3^)	Equation	R^2^	LWD (g/dm^3^)	EWD (g/dm^3^)	Average percentage difference
*Lycium europaeum*	7	664± 37.30	717.59± 24.18	698.09 ± 4.06	y = -0.1926x + 836.18	0.62	735.55± 12.96	704.81± 21.90	5.2± 1.87
*Lycium schweinfurthii*	7	709± 38.54	728.95± 50.10	751.12 ± 59.86	y = 1.2553x - 164.04	0.75	780.17± 32.48	750.20± 29.60	3.6 ± 1.86
*Calligonium comosum*	7	704± 30.82	771.58± 41.90	847.5± 40.45	y = 1.0762x + 12.585	0.82	775.72± 21.75	695.47± 38.84	11.8 ± 5.51

LWD, latewood density; EWD, earlywood density; Average percentage difference =, ((LWD-EWD)/EWD) *100.

### 3.3 Vessel traits

**Figure 6:**
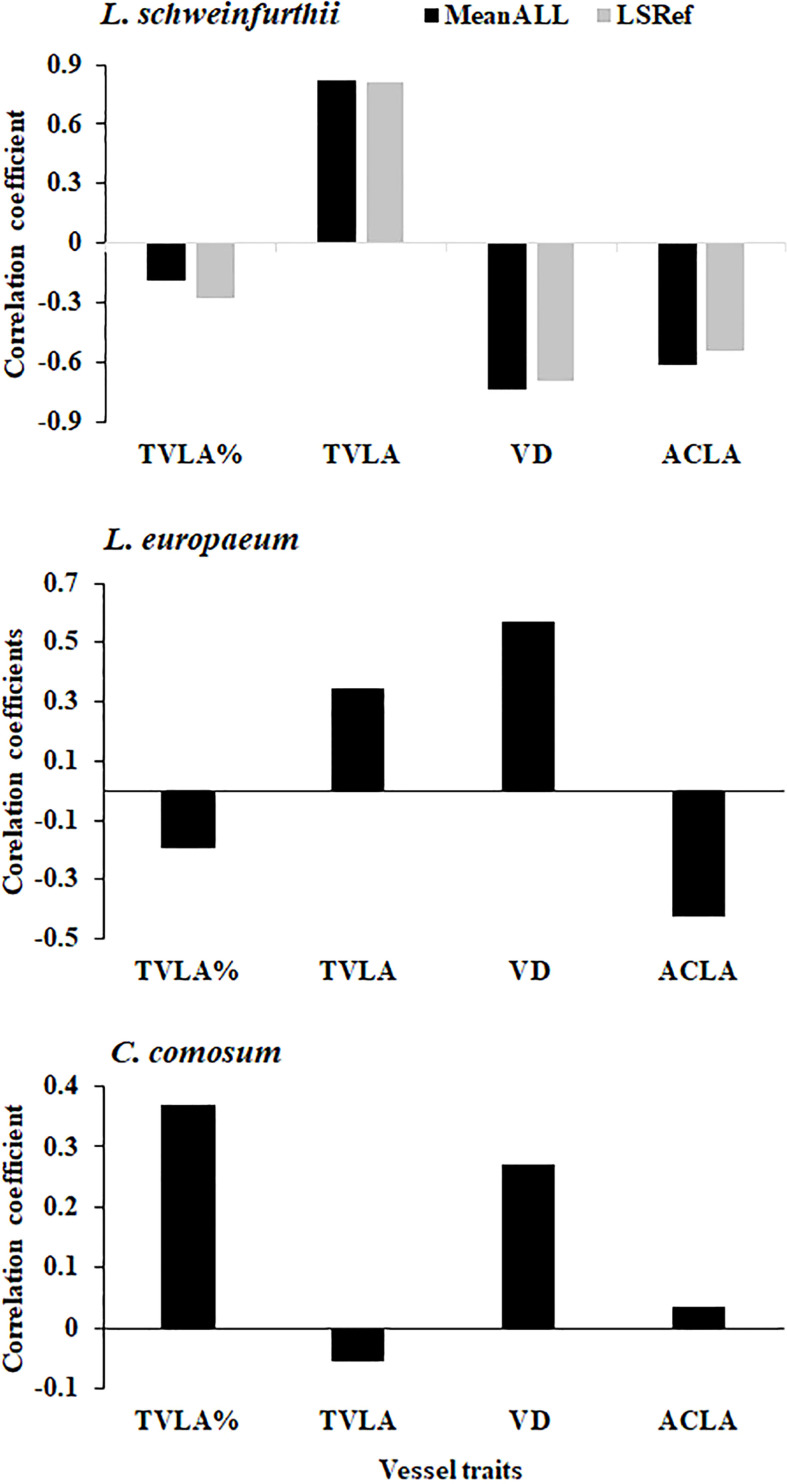
Correlation between the detrended tree ring widths and the main vessel traits of the studied species. VLA= vessel lumen area %, MTVLA, mean total vessel lumen area (µm^2^); VD, vessels density (no. mm^-2^); ACLA, Average cell lumen area (µm^2^).

The main traits of vessels for the studied species are shown in **Table 2**. The total lumen vessel area% was 8.96, 7.13, and 6.06 for *L. schweinfurthii*, *L. europaeum*, and *C. comosum*, respectively. The Mean Total Lumen Vessel Area (TVLA, µm^2^) in *L. europaeum* was ≈1.5 times higher (0.7469 mm^2^) compared to other species. On the other hand, the vessel density (VD) in *L. schweinfurthii* (108.84 N/mm^2^) and *L. europaeum* (157.10 N/mm^2^) was about 6-8.8 times higher than that in *C. comosum*. In contrast, *C. comosum* had an average Cell Lumen Area (ACLA, 66.70 µm^2^) that is > two-fold of that recorded for *Lycium* spp. This was apparent in the wide lumens of the earlywood and latewood vessels in *C. comosum* compared to the *Lycium* spp. that have extremely narrow vessels in latewood. There were significant annual variations in some vessel traits for *L. schweinfurthii* (TLVA%, TLVA, and VD), *C. comosum* (TVLA% and TVLA), but not for *L. europaeum*. It is worth noting that the total wall area % for the three species was around 80, while the average lumen vessel perimeter was three-fold higher in *C. comosum* than in *Lycium* spp. (data not shown). The correlation between detrended vessel traits and tree-ring index (TRI) showed a significant positive correlation in *L. schweinfurthii* with MTVLA but a significant negative correlation with VD and ACLA (**Figure 6**). TRI in *L. europaeum* correlated positively with MTVLA and VD but negatively with ACLA. On the other hand*, C. comosum* correlated positively with TVLA%. The relationships between TRI and vessel traits in *L. europaeum* and *C. comosum* were mostly weak (i.e., r < 0.5).

**Table 2:** Mean, maximum, and minimum values of the vessels’ characteristics of the studied species.

Species	Total Lumen Vessel Area %	Total Lumen Vessel Area (µm^2^)	Mean Vessel Density (N/mm^2^)	Average Cell Lumen Area (µm^2^)
Lycium schweinfurthii				
Average	8.96	493559.81	108.84	29.04
Max.	11.27	911235.20	153.41	34.16
Min.	7.12	158396.65	86.91	25.21
F-value	2.89**	2.53**	6.52***	1.50
Lycium europaeum				
Average	7.13	746924.17	157.10	35.11
Max.	9.78	1141585.21	224.94	40.9
Min.	4.27	392181.29	57.11	31.55
F-value	0.56	1.15	0.33	0.32
Calligonum comosum				
Average	6.06	438713.21	17.82	66.70
Max.	13.89	898610.03	24.72	88.67
Min.	3.06	137119.36	13.00	52.91
F-value	8.33***	5.38***	1.76	1.84

F-value and significance for the annual variations in the vessel traits are shown. *** p< 0.001, ** p< 0.01.

### 3.4 Radial growth and chronology

**Figure 7:**
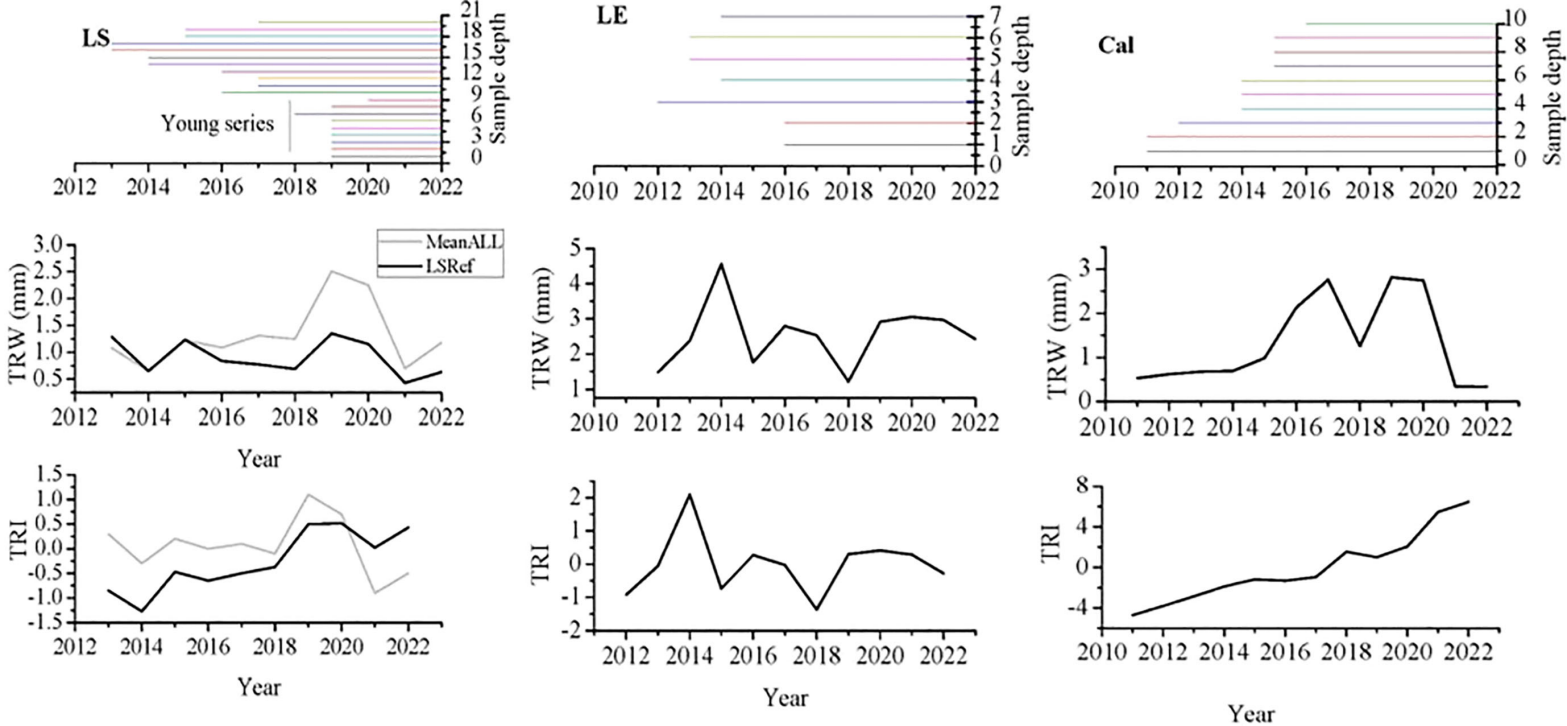
Tree ring widths (TRW, mm) and indices (TRI) of the reference chronologies of *L. schweinfurthii* (LS, and the mean of all sampled trees “MeanAll”), *L. europaeum* (LE) and *C. comosum* (Cal).

We found that the ring borders of most of the collected samples are distinct with few variations. After measuring the ring widths of different time series and analyzing them using the proper above-mentioned software programs, we had to exclude non-correlated series (examples are shown in **Supplementary Figure S2)** although they had no apparent problems. We ended with 7 trees (10 series) for *L. schweinfurthii*, 7 trees (7 series) for *L. europaeum*, and 6 trees (10 series) for *C. comosum* to build the mean site chronology for each. When we compiled all the correlated series of *L. schweinfurthii*, we ended with15 trees (20 series) (**Table 3**; **Figure 7**). The resulting raw chronology covers the years 2013-2022 for *L. schweinfurthii*, 2012-2022 for *L. europaeum*, and 2011-2022 for *C. comosum*. The ring widths were 0.9, 2.55, and 1.33 mm/year for *L. schweinfurthii*, *L. europaeum*, and *C. comosum*, respectively. The mean series intercorrelation was 0.746, 0.564, and 0.683 for *L. schweinfurthii*, *L. europaeum*, and *C. comosum*, respectively. The EPS values ranged from 0.72 to 0.80, while the SNR ranged from 9.1 to 21.5. Compiling all series of *L. schweinfurthii* raised the EPS value to 0.86. Since the wood samples of *L. schweinfurthii* and *C. comosum* were collected from the same site, the correlations between their raw and detrended reference chronologies were 0.44 and 0.74, respectively (data not shown).

**Table 3:** Statistical analysis of the reference chronologies of *Lycium schweinfurthii*, *Lycium europaeum*, and *Calligonium comosum*.

species	No. of trees (series)	Time span (years)	Mean ring width (mm/year)	Mean series intercorrelation coefficient	EPS	SNR	Average mean sensitivity	Auto-correlation
*Lycium schweinfurthii*_LSRef	7 (10)	2013-2022	0.90	0.746	0.75	20.6	0.530	0.126
*L. schweinfurthii* _All mean	15(20)	2013-2022	1.45	0.65	0.86	27.9	–	0.16
*Lycium europaeum*	7 (7)	2012-2022	2.55	0.564	0.72	9.1	0.493	0.020
*Calligonum comosum*	6 (10)	2011-2022	1.33	0.683	0.80	21.5	0.634	0.216

### 3.5 Climate-growth relationships

**Figure 8:**
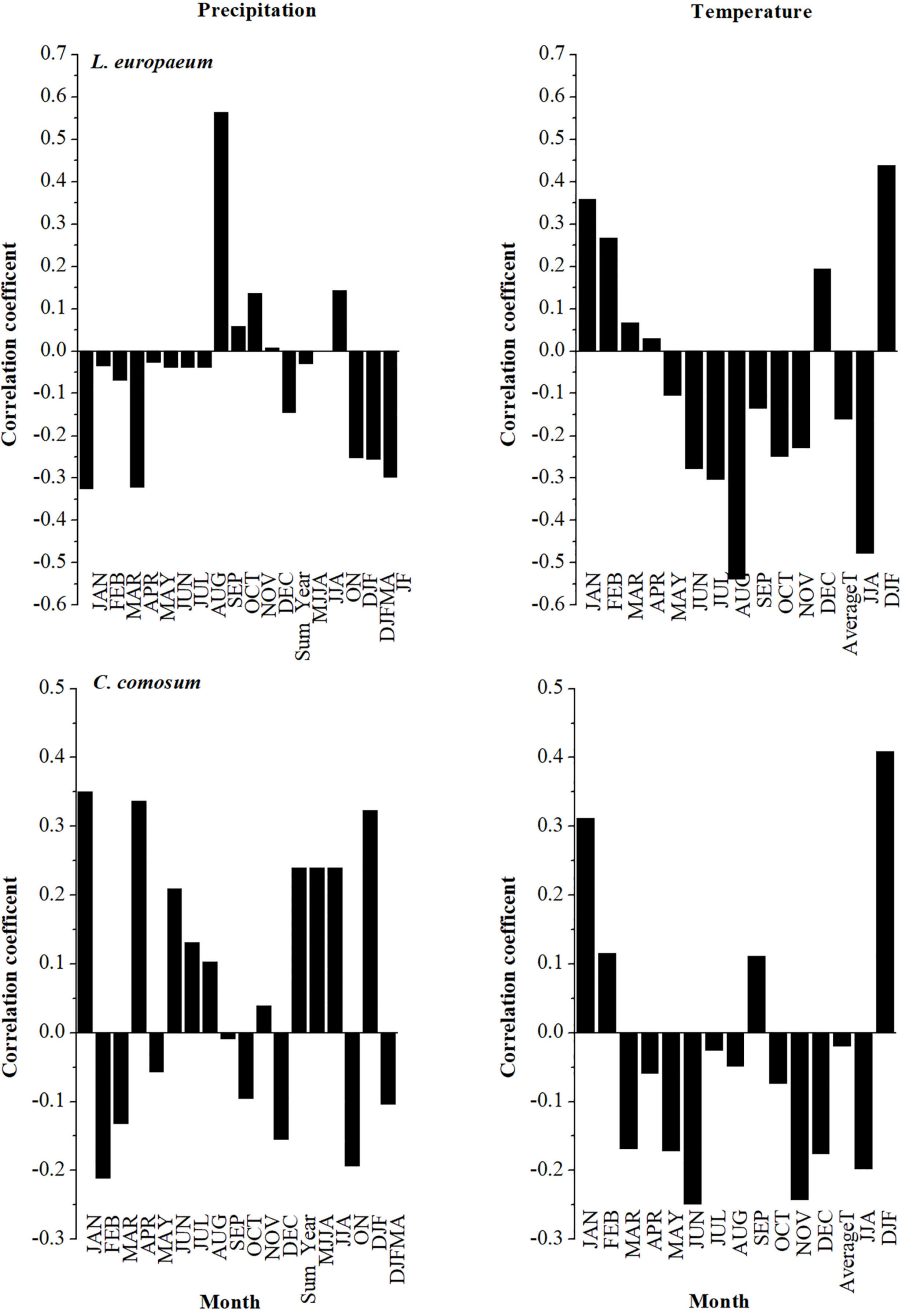
Correlation between the detrended reference chronology and Mean All data of *L. schweinfurthii* with current season monthly total precipitation (mm) and monthly Temperature (°C).

**Figure 9:**
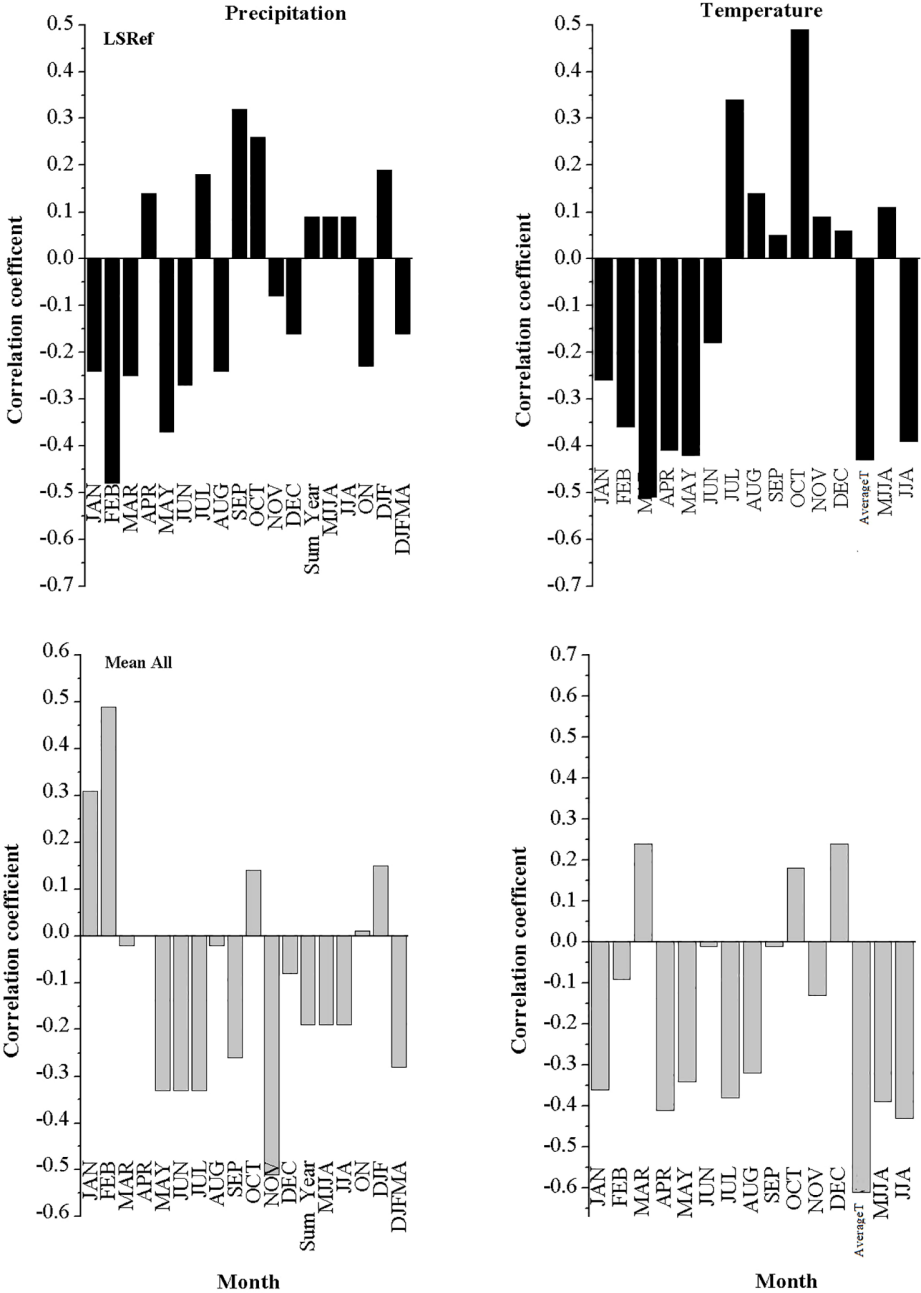
Correlation between the detrended reference chronology of *L. europaeum* and *C. comosum* with the current season’s monthly temperature and total monthly precipitation.

The correlation analysis between the studied species chronologies and climate data is shown in **Figures 8**, **9**. It was revealed from the results that *L. europaeum* and *C. comosum* had simpler and clearer correlations with climate data compared to *L. schweinfurthii*. The relationships between the radial growth of the studied species and the climate variables were weak to moderate but mostly not significant (i.e., r < 0.7). There were general positive correlations between the current season temperature during January and the period from December to February (DJF) and the radial growth of *L. europaeum* and *C. comosum*. Besides, *L. europaeum* had an overall negative correlation with summer temperature (August and JJA). On the other hand, *C. comosum* had a positive correlation with current season precipitation during January, April, and DJF months compared to *L. europaeum* and *L. schweinfurthii* which had a positive correlation with precipitation in September. *L. schweinfurthii* reference chronology and “Mean All” data showed a general negative correlation with temperature most of the year. This negative correlation was more obvious with average temperature.

## 4 Discussion

### 4.1 Wood anatomical and densitometry characteristics

Seasonal fluctuations of climatic factors are the main controlling factors that enhance the formation of distinct rings in the wood of tropical and sub-tropical plants (Rozendaal and Zuidema, 2011; Farahat and Gärtner, 2021; Gärtner and Farahat, 2021). In tropical and subtropical regions, some plants can form distinct ring boundaries (Quesada-Román et al., 2022). This may be enhanced by low or absent precipitation (Rozendaal and Zuidema, 2011) or experiencing a dry season coupled with high temperature and maybe leaf shedding that leads to cambial dormancy (Tarelkin et al., 2016; Farahat and Gärtner, 2021). In the present study, the stem microsections helped in showing well the ring boundaries in the studied trees. As well, they helped in well recognition between density fluctuations if any, and ring boundaries. That was not the case in some Amazonian trees that were explored for their ring formation where microscopic sections did not help in well recognition of growth rings (Rodriguez et al., 2022). A few samples (1-2 samples per species) had indistinct ring boundaries in one or two positions or had stain deposits in the wood that prevented the recognition of the ring borders properly. In the species studied, ring boundaries were distinct mainly by flattened fibers followed by aggregations of earlywood vessels of the adjacent rings. Moreover, clear changes in the size of latewood to earlywood vessels due to the semi-ring porous character of the rings helped more in the accurate determination of ring boundaries. These results confirm the previously published anatomical information on *L. europaeum* (Carlquist, 1992) and *C. comosum* (Neumann et al., 2000; InsideWood Database: https://insidewood.lib.ncsu.edu/).

The bulk wood density of wood for the studied species is considerably higher than average when compared to the global average of wood density (613 g/dm^3^, range: 0.08-1.39 g/dm^3^) in the Global Wood Density Database (https://opendata.eol.org/dataset/global-wood-density-database/resou). Meanwhile, the average percentage difference in wood density between LWD and EWD for the studied species was enough to differentiate macroscopically and microscopically the ring borders. Wood density correlates with the drought resistance and growth rate of the species (Hacke et al., 2001). In addition, hydraulic conductivity would decrease as wood density increased (Searson et al., 2004).

Despite the low number of measured samples, it is apparent that X-ray wood density can be used in the identification of the distinctness of growth ring boundaries in the studied plants. In the present study, there were obvious variations in the density of latewood compared to earlywood despite the presence of a few density fluctuations in some samples that entail more caution during the examination of results. The presence of earlywood vessels of the next ring assembles in a reasonable density after the ring border leads to identification of the late- and earlywood of the ring. These results are in line with previous studies on tropical and subtropical trees (e.g., de Oliveira et al., 2011; Andrade et al., 2017; Rodrigues et al., 2022).

### 4.2 Vessel traits

The wood of desert species appears to be well adapted to safety as it is to effective water transportation. This trade-off or plastic strategy is provided by numerous, narrow vessels and high values for maximum vessel diameter, respectively (Baas et al., 1983). The vessel implosion resistance may be increased by either the decreased vessel lumen diameter or the increased wall thickness alone or in combination (Cocozza et al., 2011). In the present study, *Lycium* spp. had smaller vessels and higher vessel densities than *C. comosum* which may reflect their adaptation to arid conditions compared to *C. comosum*. The presence of low vessel density but bigger vessels and average cell lumen area in *C. comosum* may increase the hydraulic conductance of the species but on account of their safety, i.e., higher susceptibility to cavitation. The xylem’s lumen diameter and vessel frequencies per cross-sectional area are features that are tremendously varied between species and are partially heritable and plastic (Al-Khalifah et al., 2006). The presence of different vessel densities for *L. schweinfurthii* and *C. comosum* which were collected from the same site (dune habitat) may reflect variations in their root depth and access to groundwater between the two species. Meanwhile, the presence of high VD and TLVA in the wood of *L. europaeum* may be one of its adaptive traits to the drought that helps in its spatial distribution along the Mediterranean coastal habitats compared to *L. schweinfurthii*. The correlation between TRI and vessel traits in the studied species was species-specific.

### 4.3 Radial growth and chronology

As we expected, not all the collected wood samples for each species reflected the common signal at the sampling sites. This expectation is based on the multi-stemmed nature of the studied species. We ended with 40-60% of the collected wood samples being valid for further analysis. As mentioned above, 1-2 samples per species had problems with the distinctness of their ring borders at certain points in the micro-section. This does not mean that the other excluded samples for each species had any problem with the distinctness of their rings. As sprouting stems or root suckers in deserts are exposed to continuous disturbances (e.g., cutting and burning), it is expected to have young ages. We observed that the excluded samples had different individual annual growth rates although they are collected from the same location. This may be attributed to many factors, particularly the variations in the microclimate of the different sampling sites, the relationship between the clonal ramets (sprouts or aerial stems), and the parent plant. For the first factor, we suppose that many site conditions may reflect the variations in growth between the individuals of the same species at the same site. For instance, access to groundwater, soil physical structure, soil temperature, and competition. The second important reason is the relationship between the new sprouts and the parent plant, and how much assimilate or nutrients they receive. In multi-stemmed trees or shrubs, this issue is highly important because there is a distinct difference between plants developed from vegetative reproduction and seeds (Nzunda et al., 2014; Alfaro-Sánchez et al., 2020; Uni et al., 2022).

The formation of underground runners, root suckers, and stem sprouts is a strategy for plants to cope with many factors, particularly disturbances and drought (Thomas, 2014). Vegetative reproduction and storage of nutrients are two main variables in rhizomatous and sprouting plants (Li et al., 1998; Herben and Klimešová, 2020). Successful resprouting depends on stored nutrients, and this is accomplished by transferring nutrients to new resprouts instead of seed formation (Bellingham and Sparrow, 2000). Compared to non-resprouting species, resprouting species are more resistant to drought stress (Zeppel et al., 2015). To support resprouting, most resprouting species also have larger non-structural carbohydrate storage and a higher allocation to roots than shoots. It was reported that many rhizomatous plants depend on the minerals and carbohydrates imported from the linked rhizomes for the growth of developing ramets (Watson, 1990). In long-lived rhizomatous plants such as bamboo, vegetative growth is influenced by the dynamics of carbohydrates and nutrients in the clonal system (Li et al., 1998). Resprouters grow at a different rate and devote more biomass to roots than non-resprouters (Clarke et al., 2013). Also, root-sprouting species are more common in ecosystems with high levels of disturbance, where mechanical disturbance can serve as the essential trigger (Herben and Klimešová, 2020). Woody shrubs have seasonal variations in their root carbohydrate reserves, typically peaking in the late fall after leaf senescence and declining in the late spring after leaf flush (Landhäusser and Lieffers, 2002). During resprouting, non-structural carbohydrate reserves are mobilized to provide carbon substrate for all carbon-demanding tasks. These reserves will then start to decline until photosynthesis is fully able to meet all carbon demands (Smith et al., 2018).

Based on the abovementioned literature, it is normal to have such variability in the annual growth of the collected samples of the same species at different sites. Most of the trees that showed high correlations and were included in the reference chronology were from one or two close sites per location. Sites heterogeneity and disturbances are among the reasons that affect tree growth patterns and may lead to dissimilar growth (Grissino-Mayer, 2001). In the present study, the obtained mean series intercorrelations for the developed chronologies are reasonable, indicating a common signal and reflecting the year-to-year variations in growth. Moreover, these values are higher than the reported average value (0.45 ± 0.16) for intercorrelation in tropical and subtropical studies for collected trees at altitudes >1000 m a.s.l (Quesada-Román et al., 2022). However, the slightly low EPS value below the recommended threshold (>0.85, Wigley et al., 1984) showed that further sampling is advantageous to strengthen the common signal of the developed chronologies, but they do not contradict our results. This was proved when EPS jumped from 0.75 to 0.86 after adding the ring widths of young trees for the chronology of *L. schweinfurthii*. The same results were reported from many tropical tree species (e.g., Brienen and Zuidema, 2005; Espinosa et al., 2018; Rahman et al., 2018) and the statistics do not accurately reflect how responsive tree-ring data are to climate variability (Buras, 2017). In addition, because of the influence of the parent plant on the growth of aboveground stems, absolute homogenous growth should not be expected. It was interesting to see the high correlation between the detrended TRI of *L. schweinfurthii* and *C. comosum* that were collected from one site. This may reflect the effect of a common climate signal or the overall site conditions on the two species.

### 4.4 Climate-growth relationships

In this study, it is revealed that the low sample depth, which resulted from the exclusion of inappropriate samples, had a detrimental impact on the significance of the relationship between climate and radial growth of the studied species. Since the used series in the chronologies had reasonable inert-series correlation, we think that increasing the sample depth will strengthen the resulting relationships and its current significance. *C. comosum* showed weak positive relationships with temperature and precipitation during the wet season (DJF) and April (for precipitation only). While *L. europaeum* had a weak to moderate positive correlation with temperature during the wet season only (DJF). Meanwhile, *L. europaeum* had a moderate negative correlation with summer temperature (JJA) On the other hand, *L. schweinfurthii* chronology had a moderate negative correlation with temperature all over the year except for July and October. The positive correlation with precipitation in September might be a statistical artifact since the ring formation should be finalized before September (see below). Nevertheless, to be sure about the period of wood formation one should apply the pinning procedure for a more detailed analysis (Gärtner and Farahat, 2021). By adding the younger series to the chronology, it produced a site chronology with weak to moderate negative correlation with the temperature but positive correlation with January and February precipitation. There is no published data on the phenology of *L. europaeum* in Egypt or elsewhere. Although, we notice that *L. europaeum* has a bit longer vegetative stage and shorter withering stage compared to *L. schweinfurthii* in the study area (own observation).

To understand the nature of these correlations, we should understand first what is controlling the wood formation in the tropical and subtropical regions. In addition to the relationship between plant phenology and wood formation. Wood is formed by the cambial cells in the tree stems (Funada et al., 2016). In most of the world’s regions including tropical and Mediterranean climates, the cambial activity of trees exhibits seasonal cycles of activity and dormancy, recognized as annual periodicity (De Micco et al., 2016). The development of tree stems, and the amount of wood produced are both influenced by the cambial activity’s periodic rhythm. It also indicates how well trees adapt to their surroundings, such as their ability to withstand the cold of winter in cool or temperate climates (Begum et al., 2018) or their ability to coordinate with seasonal leaf growth (Kitin and Funada, 2016; Gärtner and Farahat, 2021). Phenology or cycles of plant growth (phenophases) are linked with environmental factors. Currier and Sala (2022) reported that for mid and high-latitude ecosystems, rising temperatures already have phenological effects and lengthened the growing season. For the 40% of the land surface that is covered by drylands, however, precipitation change will be the primary factor in phenological change.

The available literature on *C. comosum* under the Mediterranean climate showed that vegetative growth lasts from the end of February to early May while flowering and fruiting lasts from March to early May. Seed dispersal starts from May to mid-July then the shedding of green branches during summer (July-August). The appearance of branches is in January (the coldest month). Inactive phenology was recorded from September to end-January. Limitation in water availability during the dry and hot season leads to a significant decrease in the produced number of branches per plant by about 73% (Dhief et al., 2009, **Supplementary Figure S2**). On the other hand, Beshara (2019) reported that the vegetative stage of *L. schweinfurthii* lasts from mid-September to mid-May with maximum vegetative growth from November to April (90%), while the maximum flowering and fruiting were obtained in November and April months in a bimodal pattern. The minimum flowering and fruiting were recorded in January, February, and March. Maximum withering was happening from May to October i.e., from summer to early autumn (**Supplementary Figure S2**). According to these phenological stages, the plants should use part of the stored carbohydrates and the newly formed assimilates to form wood before the flowering and fruiting stages. This could be obvious in the case of *C. comosum* which correlates positively with temperature and precipitation during the vegetative stage and before the peak of flowering in April and starting of fruiting. In *Lycium* spp., the peak of the vegetative stage (November-April, 8 months) is long enough to save the needed assimilates for building wood first and then flowers and fruits in April. It is noticeable that in these two species, the maximum flowering and fruiting percentages were reached either before or directly after the vegetation peak. It is interesting to observe also that the first flowering and fruiting peak in September was preceded by a long withering stage (May-October) according to Beshara (2019).

We can hypothesize that a decline in flowering and fruiting at the beginning of the vegetation peak may allow plants to build wood first before the second peak of flowering and fruiting. Moreover, the formation of flowers and fruits after the withering stage should be supported by carbohydrate reserves. Flowering is the most expensive phenological stage and flowers are stronger sinks than fruits (Dovis et al., 2014). Our understanding of non-structural carbohydrate (NSC) dynamics in plants is fundamentally based on the relationship between NSC and phenology. It was reported that the highest starch values were found in below-ground reserve organs, and their seasonal patterns imply that starch accumulates to support subsequent growth or metabolism when plants are dormant (Martínez-Vilalta et al., 2016). Low seasonal variability in starch and soluble sugars was a distinguishing feature of Mediterranean ecosystems. In all organs, soluble sugars displayed notable patterns, peaking in the middle of the summer. With a minimum in the spring and a high in the late summer, total NSC also demonstrated seasonal variation belowground (Martínez-Vilalta et al., 2016).

### 4.5 Conclusions, limitations, and further recommendations

The studied species had distinct ring borders but with variable annual growth rates between the individuals of the same species when collected from sites far away from each other. Despite the difficulties and presence of inconsistent growth patterns between the sampled stem discs, we succeeded in cross-dating some of them and developing short chronologies for our species. This is in line with the reported difficulties in the woods of many tropical and subtropical regions which have long been avoided by dendrochronologists (Pearl et al., 2020; Quesada-Román et al., 2022). The developed chronologies for the studied species were relatively short since we dealt with multi-stemmed shrubs. The output measures for the quality of the developed chronologies are reasonable and in agreement with many studies on tropical and subtropical woody plants. X-ray densitometry can help in the precise determination of the ring borders of the studied species. There were distinct differences in the values of X-ray densitometry for late and early wood. The relationships between bulk wood density and X-ray density were relatively strong. The vessel traits reflected the adaptability of each species with the prevailing arid climate condition. Generally, temperature and precipitation during the wet season had a weak to moderate positive correlation with ring formation, while ring formation was negatively correlated with temperature the rest of the year, particularly in summer. The sample depth finally used influenced the significance of the relationship between radial growth and climate.

For further dendroecological studies on subtropical and Mediterranean woody plants, we recommend 1- conducting xylogenesis studies using micro-cores or pinning techniques, 2- sample depth should be 3-4 times more than the minimum recommended samples for trees from temperate and cold climates (15-20 sample), 3- collecting samples from close sites taking into consideration the site conditions, with enough replica for the same tree (45-100 samples) and, 4- the cultivation of trees under simulated controlled climate conditions and investigate the ring formation during the year. We think that the collection of ecological data on the phenology of woody perennials all year around and the detection of the cambial activity are two main steps to improving or understanding the growth ring formation in tropical and subtropical trees and obtaining an accurate conclusion about its growth-climate relationship. Using other multi-proxy approaches (e.g., wood anatomy, X-ray densitometry, isotopes, dendrometers, and wood chemical analysis) will likely help with some of the challenges in tropical and subtropical ecological research and other related disciplines.

## Data availability statement

The original contributions presented in the study are included in the article/**Supplementary Material**. Further inquiries can be directed to the corresponding author.

## Ethics statement

Plant materials in this study were collected and used according to national regulations. The collection and analysis of these species for research purposes do not require any special permit. All methods in our study comply with relevant institutional, national, and international guidelines and legislation. The plants were identified by the first author according to the Egyptian flora books. No vouchers for the plants were deposited at the herbarium.

## Author contributions

EF and HG: Conceptualization and design layout of the work. EF: Collected field samples, prepared, and analyzed samples and data, and wrote the manuscript. HG: Analysis of data, revising and editing of the manuscript. All authors contributed to the article and approved the submitted version.
