# Supplementary material for: Wood anatomy and dendrochronological potentiality of some woody shrubs from the southern Mediterranean coast in Egypt
Source: Front Plant Sci. 2023 Jun 28;14:1183918. doi: 10.3389/fpls.2023.1183918 (PMC10338071; doi:10.3389/fpls.2023.1183918)
Supplement: Supplementary file 1 [file DataSheet_1.pdf]

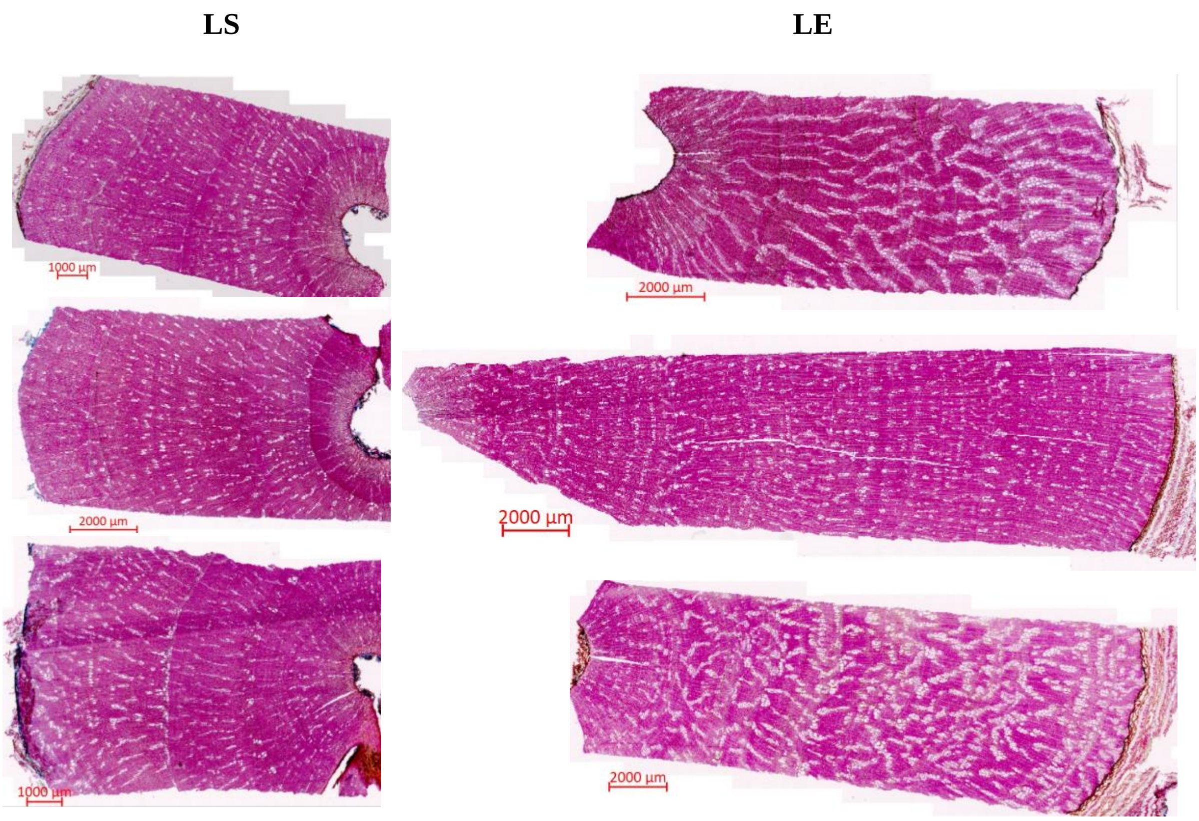

Fig. S1. Examples for excluded uncorrelated series for *Lycium schweinfurthii* (LS) and *L. europaeum.*

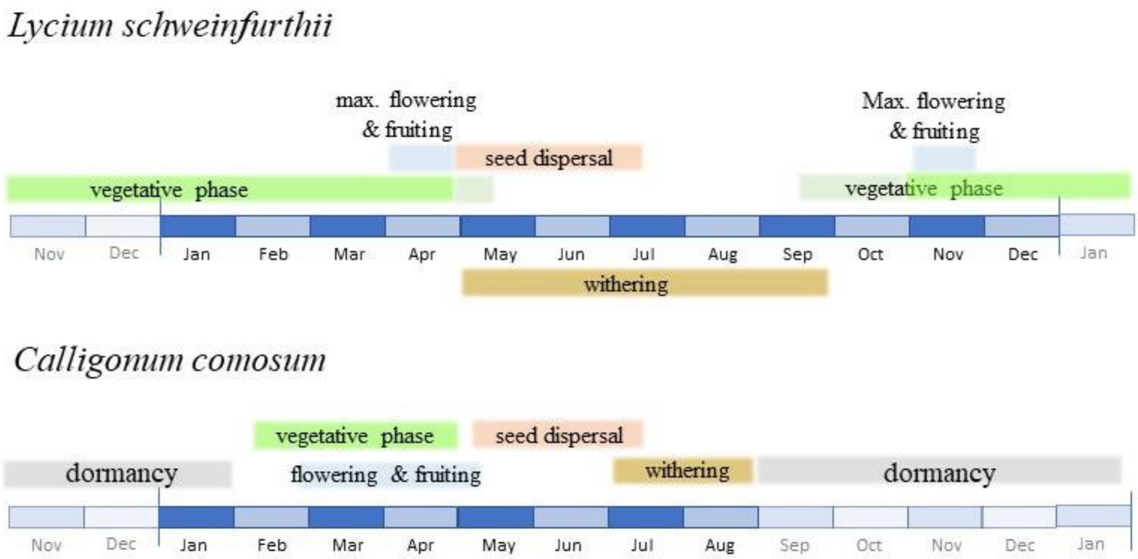

Fig. S2. Phenological stages of *Lycium schwerinfurthii* and *Calligonium comosum* along the southern Mediterranean coast (after Beshara 2019 and Dhief et al. 2009).
